# A Simple Technique to Facilitate Treatment of Urethral Strictures with Optical Internal Urethrotomy

**DOI:** 10.1155/2014/137605

**Published:** 2014-10-23

**Authors:** Konstantinos Stamatiou, Aggeliki Papadatou, Hippocrates Moschouris, Ioannis Kornezos, Anargiros Pavlis, Georgios Christopoulos

**Affiliations:** ^1^Urology Department, Tzaneio General Hospital, Zanni & Afentouli 1 Street, 18536 Piraeus, Greece; ^2^Radiology & Interventional Radiology Department, Tzaneio General Hospital, Zanni & Afentouli 1 Street, 18536 Piraeus, Greece

## Abstract

Urethral stricture is a common condition that can lead to serious complications such as urinary infections and renal insufficiency secondary to urinary retention. Treatment options include catheterization, urethroplasty, endoscopic internal urethrotomy, and dilation. Optical internal urethrotomy offers faster recovery, minimal scarring, and less risk of infection, although recurrence is possible. However, technical difficulties associated with poor visualization of the stenosis or of the urethral lumen may increase procedural time and substantially increase the failure rates of internal urethrotomy. In this report we describe a technique for urethral catheterization via a suprapubic, percutaneous approach through the urinary bladder in order to facilitate endoscopic internal urethrotomy.

## 1. Introduction

Urethral stricture causes a blocked or reduced flow of urine which can result in a range of manifestations, from an asymptomatic presentation to severe discomfort. Moreover, it can lead to serious complications such as urinary infections and renal insufficiency secondary to urinary retention. Blunt perineal trauma, urological instrumentation, chronic inflammatory disorders such as lichen sclerosus et atrophicus, and sexually transmitted diseases are the most frequent causes of strictures; a large proportion are iatrogenic [1]. Treatment of urethral strictures is often difficult because this situation is characterised by high recurrence rates and an important number of interventions are associated with poor outcomes. Currently, three different interventions are used to treat urethral strictures: dilations, optical internal urethrotomy, and open urethroplasty [1]. Treatment option depends on the type, length, and aetiology of stricture. However, the choice of treatment can be influenced to varying degrees by the simplicity of the method, the preferences of the patient, and the available accoutrements. Dilations are easy to perform in every day clinical practice; however they show the highest recurrence rates while their outcomes are the less satisfying to the patients. On the other hand open urethroplasty shows the lowest recurrence rates and its outcomes are the most satisfying to the patients [2]. Although it is the current gold standard against which the traditional treatments are compared, this technique requires skills, expertise, and equipment, often not available in the resource limited settings. For the above reasons, most patients with urethral stricture are offered optical internal urethrotomy [2]. In fact this procedure is preferred as the first treatment option by many urologists, as it is performed within short operative times either under spinal anaesthesia or under local anaesthesia. It can be also done as an outpatient procedure for the treatment of short urethral strictures [3]. Despite its popularity, internal urethrotomy shows relatively high failure rates and can be challenging and frustrating for the surgeon. In general, urethrotomy may not be suitable for long and postinflammatory strictures and potential problems like excessive bleeding, presence of blood clots, infection related lesions, and excessive damage to urethra may reduce visibility increasing thus the operative time [4]. Urethral strictures longer than 2 cm require additional operative time and often the procedure is concluded in two sessions [4].

## 2. Case Report/Description of Technique

**Figure 1:**
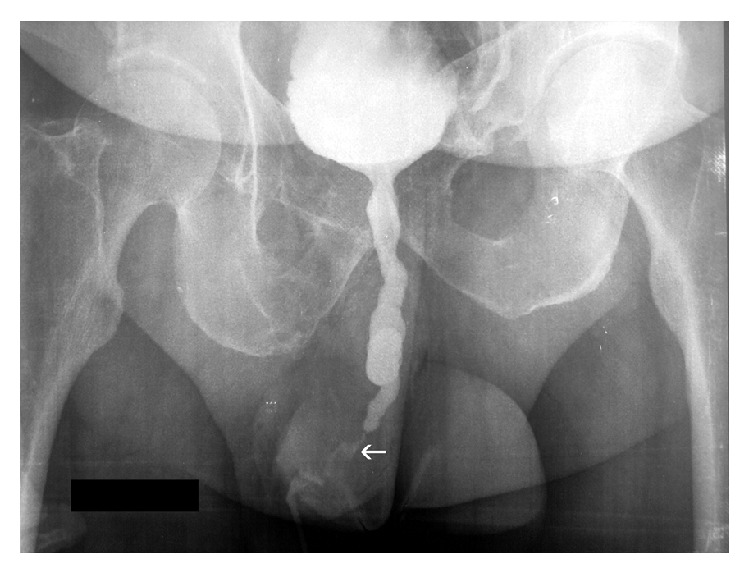
Descending cystourethrogram showing severe stenosis of the penile urethra (arrow).

**Figure 2:**
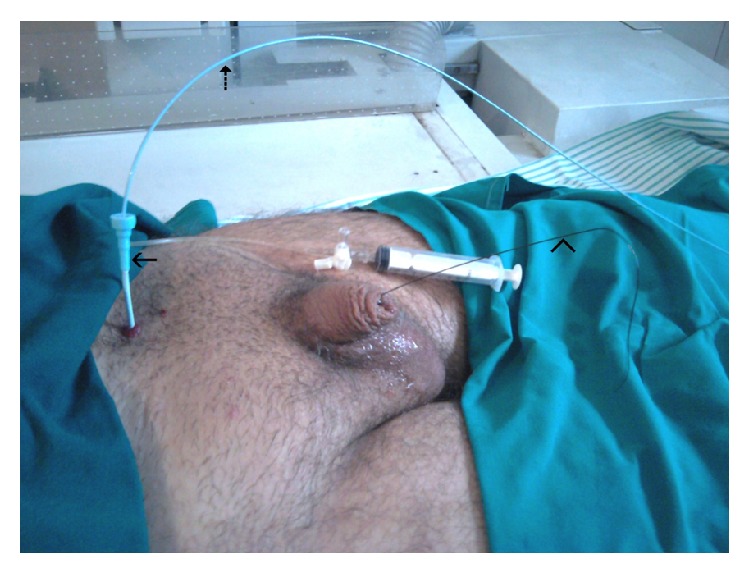
Demonstration of the technique after the replacement of the suprapubic catheter with the angiographic sheath (arrow). The angiographic catheter (dotted arrow) and the guidewire (arrowhead) have been inserted through the angiographic sheath into the bladder. The guidewire has negotiated the stenosis and has been externalized through the external urethral orifice.

**Figure 3:**
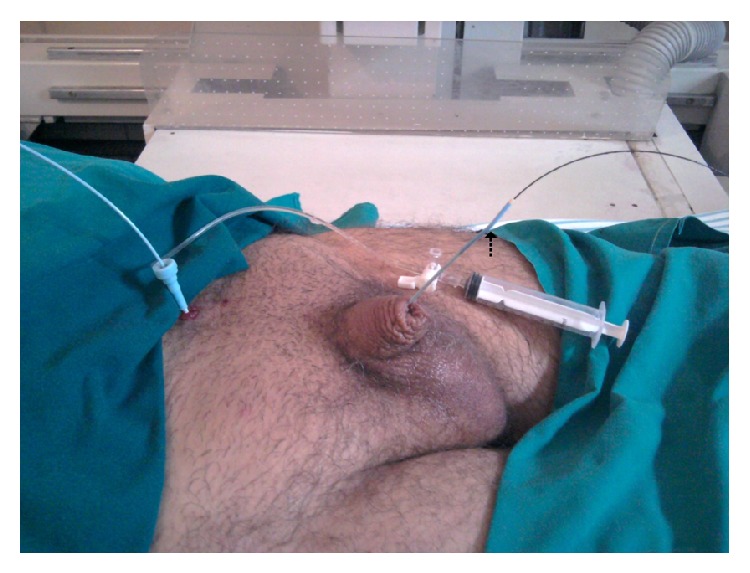
The angiographic catheter (dotted arrow) has been advanced across the stenosis over the guidewire.

**Figure 4:**
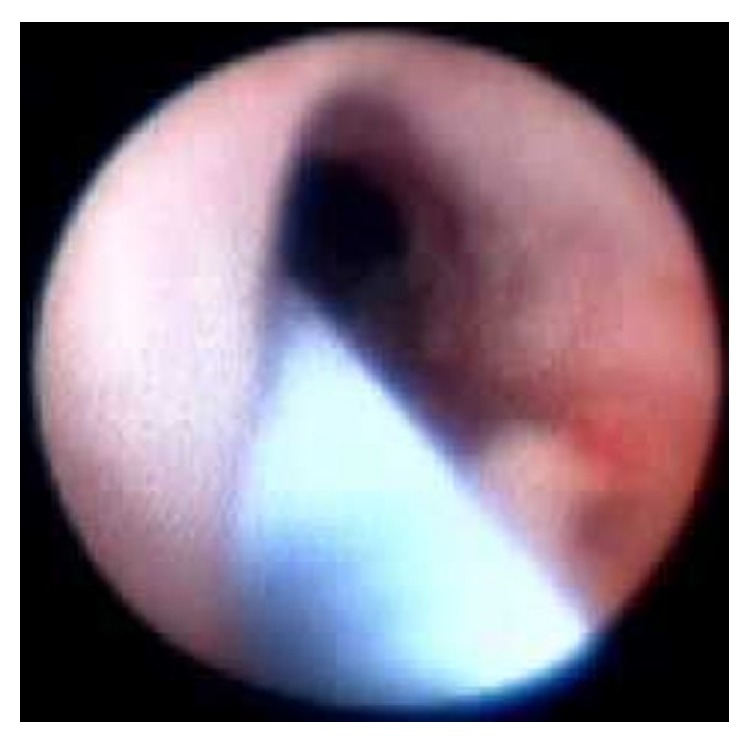
Endoscopic view of the angiographic catheter which served as a guide for the urethrotome.

A 35-year-old male patient presented to our urology department with a severe (>5 cm), tortuous stricture of the penile urethra, previously diagnosed by descending cystourethrogram (Figure 1). A suprapubic catheter was in place. Via suprapubic cystostomy, the urinary bladder was filled with 300 millilitres of diluted iodinated contrast (contrast/normal saline: 1/3). A 0035′′ J-tip standard angiographic guide-wire was inserted into the urinary bladder through the suprapubic catheter. The latter was removed and exchanged with a short (11 cm), 5-French angiographic sheath. The angiographic guidewire was subsequently withdrawn and an angiographic catheter loaded with a hydrophilic, J-tip guidewire was inserted into the bladder. Under fluoroscopy the catheter-guidewire combination was guided towards the proximal urethral orifice and subsequently into the urethra. The hydrophilic guidewire was used and finally advanced through the penile urethral orifice (Figure 2). The angiographic catheter was subsequently advanced through the stenosis, over the guidewire (Figure 3). The hub of the catheter was cut off and the vascular sheath as well as the guidewire was removed. The posterior part of the catheter was stabilized on the abdominal wall with a suture. The patient was then taken to the operating room and he was placed in lithotomy position under spinal anaesthesia. The guidewire was now placed antegrade through the angiographic catheter in order to facilitate the passage of the angiographic catheter through the working channel of the rigid urethrotome. By keeping the angiographic catheter stretched, the instrument was inserted and guided to the face of the stricture. The stricture was cut at the 12 o'clock location along the entire stenosis (Figure 4). Upon completion of the internal incision(s), the instrument was withdrawn and an appropriately sized Foley catheter was inserted through the repair into the urinary bladder. Hospitalization lasted 2 days and the patient kept the catheter a few days.

## 3. Discussion

Internal urethrotomy has advantages of ease, simplicity, speed, and short convalescence. However, success rates vary and long term results are generally low. In the short term (less than 6 months), success rates are 70 to 80 percent. After one year, however, recurrence rates approach 50 to 60 percent and by five years, recurrence falls in the range of 74 to 86 percent [4]. Although different studies have proposed different etiologies as poor responders to optical internal urethrotomy, technical and anatomical factors such as reduced visibility during the operation and stricture length are uniformly recognised as predictors of recurrence [5]. Other factors associated with treatment failure are the perioperative urinary infection, the presence of periurethral fibrosis (spongiofibrosis), and stricture etiology [5]. No visible orifice on the face of the stricture and extremely narrow, tortuous urethras predispose to urethral injury, false passage, and development of fistula. Injury increases the recurrence rate significantly (from 28% if uninjured to 72% if injured) and concomitantly the need for more procedures [6]. The stricture length has been also shown to be directly proportional to treatment failure. Pansadoro and Emiliozzi demonstrated high recurrence rate for strictures greater than 1 cm. In their study, the success rate was 71% for strictures shorter than 1 cm compared to 18% for longer strictures [7]. Retrograde instillation of methylene blue through the suprapubic catheter and/or antegrade advancement forwarding of ureteral stent are usually used in order to visualise the orifice of the structured urethra or to guide the urethrotome through a tortuous urethra, respectively. Both tricks offer limited help. In contrast, the retrograde placement of angiographic catheter safely guided the rigid urethrotome through the narrowed urethra to the bladder neck. The repeatability of the method was tested in two additional patients. No urethral injury, false passage, and development of fistula occurred in none of the three patients to whom we performed the suggested tip. Although all three patients had long and tortuous strictures (the longer was 5 cm), the operative time was brief (median 16 min). None of the patients required urethral dilatation in a mean follow-up period of 6.5 months. Of note, in two out of the three cases strictures were anterior involving bulbar urethra, while the remaining was in penile. The location of the stricture does not seem to cause difficulty to the placement of the angiographic catheter; however, it has been proved as a strong predictor of stricture recurrence in many studies [7].

## 4. Conclusion

Technical and anatomical factors such as reduced visibility during the operation and stricture length render optical internal urethrotomy difficult. The retrograde placement of angiographic catheter can safely guide the rigid urethrotome through the narrowed urethra to the bladder neck facilitating thus the procedure.
